# Detecting changes at the leading edge of an interface between oceanic water layers

**DOI:** 10.1038/s41467-019-12621-8

**Published:** 2019-10-14

**Authors:** Qunshu Tang, Vincent C. H. Tong, Richard W. Hobbs, Miguel Ángel Morales Maqueda

**Affiliations:** 1 CAS Key Laboratory of Ocean and Marginal Sea Geology, South China Sea Institute of Oceanology, Guangzhou, China; 2 Innovation Academy of South China Sea Ecology and Environmental Engineering, Chinese Academy of Sciences, Guangzhou, China; 3 Department of Earth Sciences, University College London, London, UK; 4 Department of Earth Sciences, Durham University, Durham, UK; 5 School of Natural and Environmental Sciences, Newcastle University, Newcastle upon Tyne, UK

**Keywords:** Ocean sciences, Physical oceanography

## Abstract

Many physical phenomena in the ocean involve interactions between water masses of different temperatures and salinities at boundaries. Of particular interest is the characterisation of finescale structure at the marginal interaction zones of these boundaries, where the structure is either destroyed by mixing or formed by stratification. Using high-resolution seismic reflection imaging, we present observations of temporal changes at the leading edge of an interface between sub-thermocline layers in the Panama Basin. By studying time-lapse images of a seismic reflector between two water boundaries with subtle differences, we provide empirical constraints on how stratified layers evolve. The leading edge of this reflector, which is characterised by a gradual lateral decrease in vertical temperature contrast ($|\Delta T|$), increases in length over ~3 days coupled with an increase in $|\Delta T|$. A critical mixing state, in which turbulent diffusion is gradually replaced by double-diffusion as the dominant mixing process, is thus revealed.

## Abstract (web summary)

The marginal interaction zones of oceans are understudied. Here, the authors analyse seismic observations of temporal changes at the interface between thermocline layers in the Panama Basin, that reveal a critical mixing state in which turbulent diffusion is gradually replaced by double-diffusion as the dominant mixing process.

## Introduction

Seismic oceanography has revolutionised the study of the internal structures and physical properties of ocean waters over scales ranging from the order of 0.01–100 km^1^. Using conventional marine multichannel seismic reflection data through seismic imaging, inversion, and spectral analysis methods, various oceanic features, such as mesoscale eddies^2,3^, sub-mesoscale fronts^1^, finescale internal waves and turbulence^4^, can be explored and, together with hydrographic observations, provide on-going constraints on the fundamental physical processes within the oceans. Continuous seismic reflections, reveal the stratification of the ocean and allow us to understand the internal structures of the water column, the development of water layers, their spatial extents, and the dynamic processes occurring in and between them.

Ocean interfaces with relatively abrupt vertical changes in temperature and salinity, supporting internal waves and controlling vertical transports, are fundamental to understanding global ocean mixing^5–7^. Thermocline studies have investigated both mechanically driven and double-diffusive diapycnal mixing within the framework of the global overturning circulation^8–11^. An implication of mixing is that there has to be a perpetual regrowth of interfaces or restratification of the water to balance the effects of turbulence that destroys them^12,13^. Because this process of restratification is poorly understood, monitoring and measuring the changes in the spatial extent of the stratified layers with resolution sufficient to map subtle changes is crucial to progressing the understanding of the underlying physical processes. Further, these observations are vital to provide empirical constrains for numerical modelling of thermohaline restratification^7^. However, proposed 1D-based sheet-and-layer models based on sparse conventional hydrographic profiling observations of the fine-structure do not provide adequate constraints on the horizontal extent or temporal evolution of the processes of thermohaline layering, fluctuation, and mixing^14^.

To overcome this limitation, we use time-lapse data from an underway seismic survey in the Panama Basin. Most of the previous seismically detected ocean phenomena are usually defined by ensembles of seismic reflections^1,2,15^. No study that has hitherto provided analysis of a specific individual reflector at a water interface, especially towards the tip of the reflector where a mappable interface is being created. From the seismological viewpoint, the reflector ends because the signals become too weak to detect above the ambient noise. The strength of reflection is a function of both the magnitude of change, mainly of temperature, between the neighbouring water layers and the gradient of that change^16^. Though time-lapse observations of water boundaries are technically possible they are rare as during a seismic survey, ships rarely go back to the same place to take measurements within an appropriate time window suitable to capture the variable and multi-scale ocean features. It is also difficult to track a specific reflector to decipher its evolutionary history without any other concurrent evidence.

In this paper, thanks to the seismic survey plan we were able to acquire a time-lapse image that has allowed us to capture and study the evolution of a reflector and its leading edge in the tropical ocean. Our study provides quantitative constraints on the spatially varying properties of the vertical temperature contrast (Δ*T*), buoyancy fluxes, and the temporal evolution of the interface’s leading edge. This approach not only reveals a critical mixing state in which turbulent diffusion is gradually replaced by double-diffusion as the dominant mixing process, but also demonstrates the value that time-lapse seismic images provide to study spatio-temporal changes with high lateral resolution of interaction zones between water masses.

## Results

### Seismic reflection of the water interfaces

**Fig. 1:**
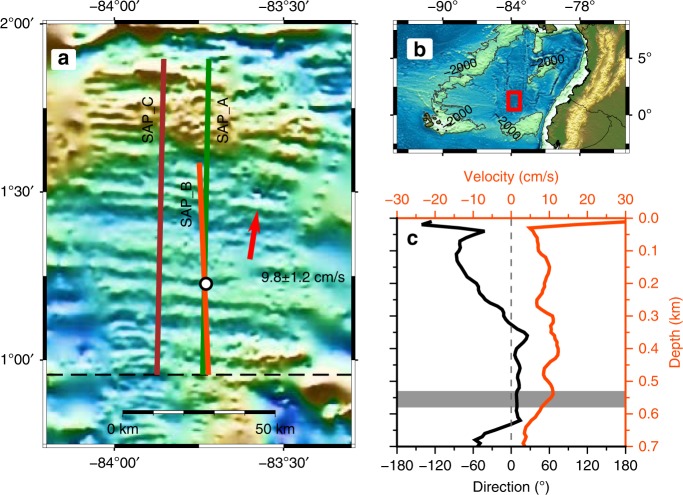
Seismic and oceanographic observations in the Panama Basin. **a** Three north-south orientated seismic profiles (orange: SAP_B, green: SAP_A; brown: SAP_C). SAP_B and SAP_A are almost co-located but with a time gap of ~3 days (Fig. 2d). SAP_A and SAP_C were acquired on parallel profiles separated by a distance of 15 km apart but recorded within 1 day of each other (Supplementary Fig. 1). Red arrow: the mean background current between 530 and 580 m depths from shipborne Acoustic Doppler Current Profiler (ADCP) during the seismic observation. Black circle: a CTD cast on 22 Dec 2014 shown in Supplementary Fig. 2. The dashed black line is the common reference point for the seismic profiles presented in Fig. 2. **b** Overall map of the study region in Panama Basin. **c** Average current velocity (orange) and direction (black) profiles derived from the shipborne 75 kHz ADCP. Grey band: the depth of the interface under investigation (Supplementary Fig. 5)

**Fig. 2:**
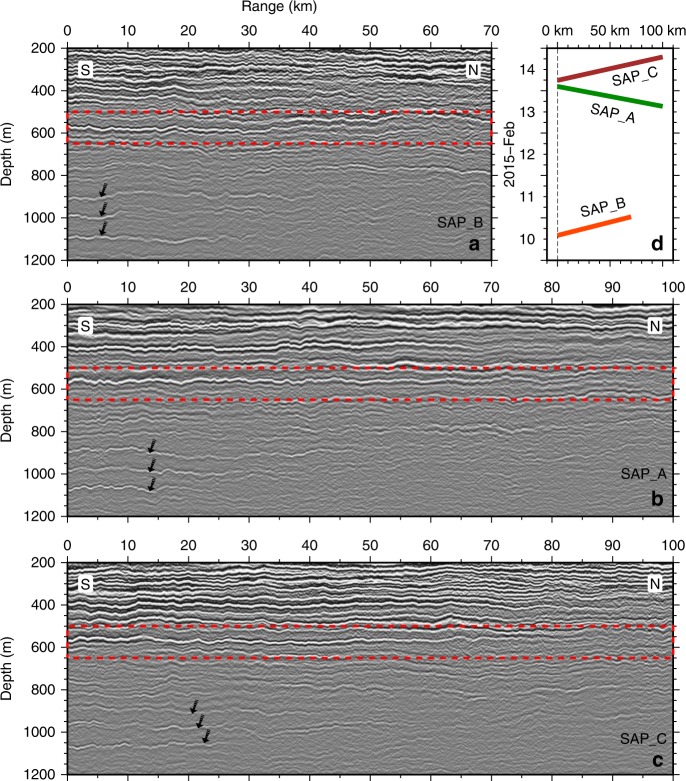
Seismic images from 200 to 1200 m depth. Three time-lapse seismic sections **a** SAP_B, **b** SAP_A, **c** SAP_C, and **d** their acquisition time. SAP_B and SAP_A are co-located but separated in time by ~3 days whereas SAP_A and SAP_C were collected consecutively but are spatially separated by 15 km. The boxes (red dashed lines) highlight the target reflections shown in Fig. 3. The consistent spatial relationship between the target reflections and the co-existing three-reflection group (black arrows) give confidence that we are tracing the same reflector on the time-lapse and spatially shifted sections

**Fig. 3:**
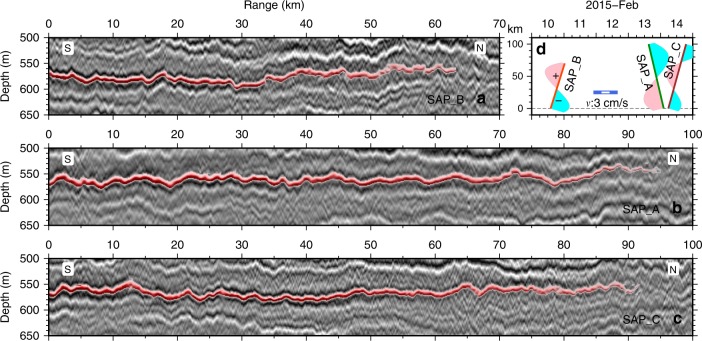
Enlargements of the target reflections (red) at ~560 m depth in Fig. 2. Three time-lapse seismic sections **a** SAP_B, **b** SAP_A, **c** SAP_C, and **d** their acquisition time and the predicted meridional component of the tidal current. The blue and pink filled areas represent the amplitude of the meridional component of the tide relative to the scale in the centre of the plot

Three seismic images show finescale structures of the water column within the equatorial Panama Basin (Figs. 1–3; Supplementary Fig. 1). Corresponding to the 1D sheet and layer model, seismic reflectors are the sheets which represent temperature interfaces, and the zones that are absent of reflections are the layers which are composed of relatively homogeneous water. The general stratification of the sub-thermocline (i.e., from the base of the thermocline to the top of the intermediate water) is presented on the seismic section along the seismic reflection profile SAP_A (Supplementary Fig. 1). Strong and continuous reflections are imaged mainly above 600 m depth, and weak and intermittent ones are imaged mainly below 600 m depth. From the calculated reflectivity of a CTD profile acquired ~50 days prior to the seismic survey, the depth of 600 m marks the upper boundary of the intermediate water. The stratification here favours the double-diffusive process of salt fingering as the density ratio $R_\rho \approx 5$ (Fig. 1; Supplementary Fig. 2; Table 1). Reflections over the deeper abyssal basin, to the southern end of the profile, are stronger and more continuous than those over the shallower mid-ocean ridge, at the northern end, even though these features are at a depth of 3500 and 2500 m, respectively.

**Table 1:** Primary values and results from seismic and hydrographic measurements of the time-lapse interface

Variable	Value	Description
$z$	~520–600 m	Target reflection depth
$L$	> 50 km (SAP_B); > 90 km (SAP_A, SAP_C)	Observed interface length
$l$	~50 km	Leading edge width
$\Delta z$	12.5 ± 2.5 m	Interface thickness
$\Delta T$	~ −0.3 °C to −0.08 °C	Temperature contrast across the interface from centre to tip by AVO inversion
$\frac{\partial \Delta T }{\partial x}$	0.45 ± 0.05 °C per 100 km	Lateral *ΔT* variation
$\frac{dx}{dt}$	13.0 (3.4 ± 1.2) cm s^−1^ (stacked); 14.2 (4.6 ± 1.2) cm s^−1^ (pre-stack)	Uncorrected (corrected) expansion velocity in x-direction
$\frac{\partial \Delta T}{\partial t}$	(1.8 ± 0.5) × 10^−7^ °C s^−1^	*ΔT* change rate
$\beta F_s$	1.5 × 10^−10^ to 0.2 × 10^−10^ m s^−1^	Buoyancy flux due to salt from centre to tip
$\alpha F_T$	0.8 × 10^−10^ to 0.1 × 10^−10^ m s^−1^	Buoyancy flux due to heat from centre to tip
$\sigma _0$	27.0 kg m^−3^	Potential density referenced to 0 dbar
$N$	3.6 × 10^−3^ s^−1^	Buoyancy frequency
$R_\rho$	~ 5.0	Density ratio
$\left( u,v \right)$	(1.5 ± 0.2, 9.6 ± 1.2) × 10^−2^ m s^−1^	Background current velocity
$\frac{dU}{dz}$	(5.3 ± 0.9) × 10^−4^ s^−1^	Vertical shear
$Ri$	47 ± 16	Gradient Richardson number

Towards the southern end of the sections, reflections become more elongated and easy to track with progressively enhanced reflection strength (Fig. 2). Two specific depths with prominent reflections can be identified. One ranges around 520–600 m depth: a single strong reflection ~50 km long on SAP_B and ~90 km long on SAP_A and SAP_C. The other is around 900–1100 m depth: three distinct reflections with near-equal vertical spacing of 100 m forming a characteristic combination on each of the seismic profiles. These reflections are highly likely to be caused by the same reflectors, as they show the same characteristics on each profile. First, they are centred at the same depth intervals with little vertical shift during the observation period. Second, their lateral variation from strong to weak and finally dying out towards the ends of the reflections is a consistent feature. And third, they have a strong amplitude associated with the relatively thick and well mixed layers above and below. Here, we focus on the single reflection at ~560 m depth for further time-lapse analysis because of its advantages in both reflection strength (high signal-to-noise ratio, SNR) and spatial continuity (reliability in tracking).

From enlarged seismic images of the tracked reflectors (Fig. 3), a vertically thin but laterally extensive interface is spreading and developing in the water modulated by internal waves along the isopycnal *σ*_0_ ≈ 27.0 kg/m^3^ (Supplementary Fig. 2a; Table 1). Its north-south extent is >100 km and its east-west extent is at least tens of kilometres from other seismic sections acquired during the same cruise (not shown here). The gradually reducing strength of the reflection amplitude towards the tip or the leading edge of the stratified interface indicates a progressive varying of the reflectivity from strong to subtle and then to undetectable with a broad transition zone of tens of kilometres.

### Temperature variations across and along the water interface

**Fig. 4:**
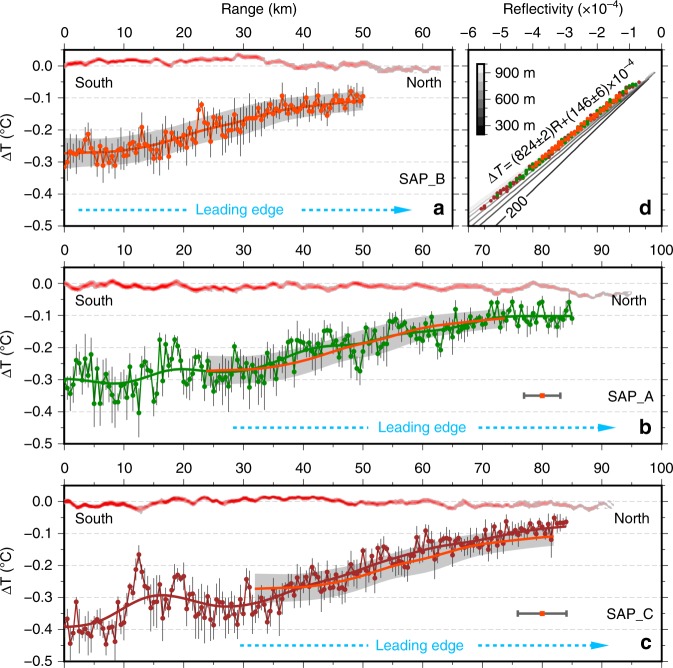
Variations of the temperature contrasts (Δ*T*) along the seismic reflectors. **a** Δ*T* (orange dots with grey uncertainty bars of one standard deviation) derived from a combined AVO and Markov Chain Monte Carlo analysis (Supplementary Figs. 3, 4) for traced interface from seismic profile SAP_B. The orange line and the grey band are the spatially smoothed Δ*T* and uncertainty, respectively. The tracked seismic reflection in Fig. 3a is also shown at the top of the panel. The blue arrow outlines the leading edge of the interface from the centre to the tip. **b**, **c** Same as (**a**) for seismic profiles SAP_A and SAP_C, respectively. The smoothed SAP_B reflection in (**a**) is displaced according to the current drifted offsets with the magnitude of the uncertainty in the location of this reflection represented by the orange dot with grey bar. **d** Plot showing the consistent and near-linear relationship between reflection coefficients and Δ*T* for all three reflections. Theoretical relationships between them at every 100-m depth are presented for reference (grey lines)

Physical properties of temperature contrast across each of the interfaces are recovered (Fig. 4) from the pre-stack seismic data using the technique of Amplitude Versus Offset (AVO; Methods), which is a prevailing geophysical approach for determining various characteristics of reflection interfaces^17^. Following the AVO analysis of the water interface by Paramo and Holbrook^18^, we improved the inversion scheme that directly inverts for Δ*T* and its uncertainty from AVO characteristics of reflectivity (Supplementary Fig. 3) using a Monte Carlo Markov Chain method (MCMC; Supplementary Fig. 4).

The inverted Δ*T* across the interface, computed every 500 m along the profiles, show relatively large (−0.3 °C) temperature differences in the south, and a uniform gradual decrease in absolute Δ*T* towards the north at a rate of about 0.45 ± 0.05 °C every 100 km (Fig. 4). Meanwhile, the relationship between Δ*T* and reflectivity shows a linear relationship (Fig. 4d), providing a fast way to estimate Δ*T* from the seismic reflectivity assuming the interface thickness is significantly less than the seismic wavelength of about 40 m. As with the reflection character, the similar Δ*T* trends of the reflection provide additional evidence that these reflections are from the same water interface. The lower signal-to-noise ratio on the pre-stack data means that the lengths of the reflection that can be tracked for AVO analysis are about 10 ± 3 km shorter than from the stacked seismic sections. A Δ*T* of ~0.08 ± 0.02 °C is estimated to be the minimum value that may be determined from AVO analysis using these pre-stack data.

### Time-lapse evolution of the water interface

**Fig. 5:**
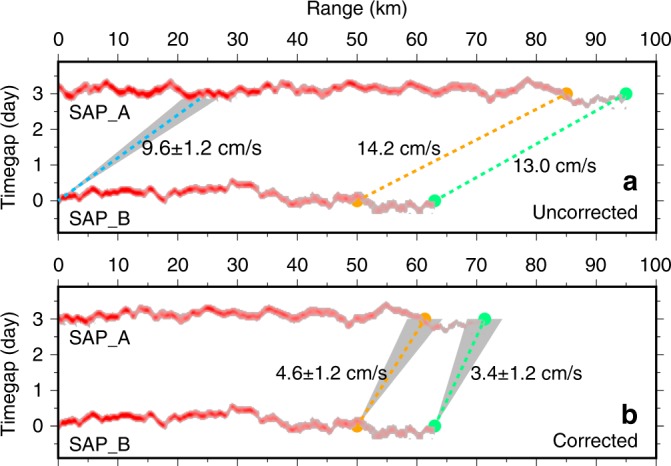
Lengthening rate estimation by removing the mean background current. **a** Observed spatio-temporal locations of the reflections (red) mapped on seismic profiles SAP_A and SAP_B. The blue dashed line represents the displacement and its uncertainty (grey) caused by the background current speed of 9.6 ± 1.2 cm/s. The yellow and green dots show the tip locations from AVO and stacked sections, respectively. The yellow and green dashed lines show their absolute movements with speeds of 14.2 cm/s and 13.0 cm/s, respectively. **b** Same as (**a**) but after the removal of the movement caused by the background current to show the estimated lengthening speeds from SAP_B to SAP_A as determined from the pre-stack data of 4.6 ± 1.2 cm/s (orange) and stacked data of 3.4 ± 1.2 cm/s (green)

The time-lapse difference seen between profiles SAP_B and SAP_A/SAP_C is a combination of advection, spatial shape of the interface and growth. It is necessary to separate these possible causes so the growth of the interface can be isolated. For the duration of the profiles shown in Fig. 3, the direction of the current at ~560 m depth, as determined from the shipborne Acoustic Doppler Current Profiler, backs from 20° to −20° as the vessel heads south along SAP-A then veers back to 20° as the vessel heads north again along SAP-C whereas the magnitude of the current remains reasonably constant at about 10 cm/s (Supplementary Fig. 5). Taking the mean, we estimate a background current of 9.8 ± 1.2 cm/s at 9.0 ± 2.1° from north, which resolves into the easting and northing components of (*u*,*v*) = (1.5 ± 0.2, 9.6 ± 1.2 cm/s), respectively (Fig. 1c; Table 1). By checking the water parcel advection of both mean current and tidal current during the seismic acquisition, we argue that the reflector’s tip was repeatedly mapped within an uncertainty of 95% confidence interval while the uncertainty by tidal excursion is insignificant (Supplementary Fig. 6). After correction for this movement caused by the mean current, considerable spatial offsets of the trackable reflection tips still exists (Fig. 5; Table 1). To account for this offset we propose that the observed interface is lengthening at rates estimated at 3.4 ± 1.2 cm/s and 4.6 ± 1.2 cm/s from the stacked section and AVO data, respectively (Table 2).

**Table 2:** List of velocity components (advection rate plus lengthening rate equals expansion rate) of the water interface shown in Fig. 5

Data	Component	Duration (h)	Velocity (cm/s)
Hydrographic	Background advection	88.03	9.6 ± 1.2
Stacked seismic	Expansion	66.00	13.0
	Lengthening		3.4 ± 1.2
Pre-stack seismic	Expansion	68.28	14.2
	Lengthening		4.6 ± 1.2

From Δ*T* variations it is not possible to directly extract the temperature change rate $\frac{\partial \Delta T }{\partial t}$ at the interface’s leading edge (Fig. 4). However, it can be derived from the lateral temperature variation rate $\frac{\partial \Delta T }{\partial x}$ and the lengthening rate $u_R = \frac{{dx}}{{dt}}$ using the relation $\frac{\partial \Delta T }{\partial t} = - u_{R}\frac{\partial \Delta T }{\partial x}$ (Methods). This gives an average increase in absolute temperature change rate of (1.8 ± 0.5)×10^–7^ °C/s as the interface matures. So, the accumulated Δ*T* change at the interface leading edge is ~0.05 °C in 3 days, which is below the detection level of ~0.08 ± 0.02 °C from the MCMC AVO analysis – the reason why it is not possible to measure the temperature change directly. Note that this temperature change rate or growth rate falls into the ranges from both numerical experiments and in situ observations for the double-diffusive staircases^19,20^.

In the above, we have assumed the role that the vertical shear of the horizontal current can play in sharpening or dulling thin fluid layers is negligible. The equation^21^ for the rate of change with time of the vertical temperature gradient is $dT_z/dt = - u_zT_x - v_zT_y - w_zT_z + F$, where a subscript denotes partial derivation with respect to the corresponding variable and *F* encompasses all turbulence contributions. The following back-of-the-envelope calculation demonstrates that the contribution of the vertical shear of the horizontal current to *dT*_*z*_/*dt* cannot realistically account for the observed changes. Indeed, for a temperature change of about 0.1 °C, as inferred, across a depth of 12.5 m (the typical thickness of the interface; Table 1) over a period of 3 days (the time separation between seismic lines; Table 1), $dT_z/dt \approx 3 \times 10^{ - 8}$ °C m^−1^ s^−1^. Given that *u*_*z*_ is on the order of 5 × 10^−4^ s^−1^ (Table 1), the horizontal temperature gradient that would be required to account fully for the estimated *dT*_*z*_/*dt* would be 6 × 10^−5^ °C m^−1^, or a temperature slope of 6 °C in 100 km, which is grossly unrealistic. Therefore, the relatively weak vertical shear of the horizontal current we observed cannot plausibly explain the observed changes in temperature contrast observed across the reflector.

### Link finescale observations with microscale processes

Finescale observations of thermocline, whether in a relatively smooth or extremely sharp gradient regime, attract much attention but direct observation normally suffers from sparse sampling^22^. This can be problematic to ascertain whether the same step/interface is being detected and thus to distinguish whether the interface change is due to growth or advection^10,23^. This difficulty in tracing finescale variations in time and space means much of the understanding on the development of thermoclines is by numerical simulation^7^. With the data presented here, we have captured a developing water interface with sufficient spatial resolution to address fundamental questions concerning the nature of this interface. Further, finescale variations of the interface are locally varying (irreversible) but globally ordered (reversible)^14^. Locally, the irregular vertical fluctuations and Δ*T* variations are modified by random internal waves or local mixing processes. Globally, its mean depth remains unchanged; its Δ*T* trends are near stationary towards the tips; and its lateral movements are consistent within the time window.

To link the finescale observation with the microscale diffusion process of the water, an estimate based on the relation between the Δ*T* and buoyancy flux due to heat/salt across the interface is inferred using an empirical parametrisation scheme in the context of a salt finger regime^24,25^ (Supplementary Fig. 7) as the density ratio $R_\rho$ ≈ 5 is consistent with the presence of salt-fingering at ~560 m depth^26^ (Supplementary Fig. 2b). The buoyancy flux due to salt gradually decreases from about 1.5 × 10^−10^ m/s to 0.2 × 10^−10^ m/s towards the tip, approximately two times higher than the buoyancy flux due to heat regulated by the flux ratio (Supplementary Fig. 7). On the one hand, the overall estimated fluxes are about one order of magnitude lower than in a typical staircase region^27^, which is consistent with the relatively high density ratio in the study region. On the other hand, the heat/salt transport by double-diffusion is increasing with the maturing of the interface, or, equivalently, is decreasing towards the tip. Compared to the numerical analysis of the competition between turbulent mixing and double-diffusion^26^, the lengthening and strengthening of the reflection’s leading edge reveals the existence of a critical state in which turbulent diffusion is gradually replaced by double-diffusion as the dominant mixing process.

## Discussion

The sub-thermocline in the equatorial ocean is free of strong current shear (Supplementary Fig. 8) or strong internal wave disturbance^28,29^. Such an environment is prone to the development of sheets and layers, such as thermal layering^30^. Hence, our observations form a representative example of a sheet-and-layer development process in a temperate ocean. However, the leading edges studied here may not be adequate to describe those found in other ocean dynamic conditions, such as those associated with eddies, staircases, interleaving, and fronts^1,15,31,32^. Results from suitably designed seismic experiments could be analysed in a fashion similar to the one presented here to investigate the spatio-temporal characteristics of the leading edges of water interfaces and the processes that control their evolution (e.g., in our study, turbulent and double diffusion).

The existence of quasi-stable, horizontally spreading water interfaces through the ocean requires a balance between the processes that cause them to grow and turbulence, which destroys them. Numerical simulations suggest a time scale for the development of interfaces from weeks to decades in an idealised system^26^. Providing constraints for these models from field observations has not been possible due to sparse spatial and temporal coverage that cannot distinguish between growth and advection. Our study provides the first robust empirical estimate on a spatial lengthening rate of ~3–4 km/day and an increasing absolute temperature change at a rate of (1.8 ± 0.5) × 10^−7^ °C/s for the development of a specific type of interface, providing a more complete picture of the evolution in space and time of these features than has been previously achieved. This result reveals the potential of time-lapse seismic observation and in situ hydrographic data to provide the links from the mesoscale to the finescale and then to the microscale processes that control how interfaces evolve.

## Methods

### Seismic data acquisition and imaging

From January to February 2015, a multichannel seismic (MCS) reflection experiment was conducted during the Oceanographic and Seismic Characterisation of heat dissipation and alteration by hydrothermal fluids at an Axial Ridge (OSCAR) Project aboard the RRS *James Cook* in the Panama Basin, eastern equatorial Pacific^33^. Among the seismic datasets, three primary transects labelled SAP_A, SAP_B, and SAP_C (Supplementary Fig. 1) are analyzed for this study. The seismic source was a tuned array of 6 BOLT guns (total volume of 1120 cu in). These guns were deployed at 8 m depth and triggered every 60 s (~140 m interval) at the pressure of 2000 psi. The seismic data, with sampling rate of 2 ms, were collected using a 360-channel streamer with 12.5 m channel spacing and 130 m minimum offset towed at 10 m depth.

A conventional seismic processing sequence is applied for imaging the water structures: geometry definition, noise attenuation, stacking velocity analysis, normal moveout, and stacking. In the first step, the geometry is defined based on the true shot locations to create the coordinates for the MCS data. Then three minor steps are carried out to enhance the signal-to-noise ratio (SNR) of the raw data, including (a) anomalous traces removal, removal of the noisy traces close to the stern and depth control points along the streamer; (b) band-pass filtering using a zero-phase 5–100 Hz filter; and (c) direct wave suppression using an eigenvector filter^34^. After common midpoint (CMP) sorting, an optimum stacking velocity profile is picked using a semblance map derived from a merge of three adjacent CMP gathers. Using the stacking velocity profile, normal moveout (NMO) corrections are applied to each CMP gather to account for offset dependent travel times and flatten all water reflections; a time variant mute suppresses distorted events at farther offsets. And finally the traces within each CMP gather are stacked to enhance SNR and create seismic section of the water column structure (Fig. 2).

### AVO reflectivity response extraction

To extract the Amplitude versus Offset (AVO) curves, additional processing procedures are applied to the pre-stack seismic data to preserve the true amplitude of the signals, which are spherical spreading compensation and hydrophone array-directivity correction^17,18^. Due to the sparse and irregular shot interval, a super-gather of 20 CMPs within a 125 m zone, which is comparable to the first Fresnel zone at the target depth 560 m, are merged to extract an averaged AVO response curve for locations spaced every 500 m. Incidence angles of <60° are used for AVO analysis because beyond that angle, the signal amplitude is distorted by the attenuation caused by a notch in the frequency spectrum from the receiver array response^18^.

We use the following relationship to compute the water reflection coefficient *R*_w_^35,36^:

1 $$R_{\mathrm{w}} = \frac{{R_{{\mathrm{sf}}}}}{{A_{{\mathrm{sf}}}}} \cdot A_{\mathrm{w}}$$

where *R*_sf_ is the reflection coefficient of the seafloor, and *A*_w_ and *A*_sf_ are the seismic reflection amplitudes of water reflector and seafloor, respectively. The seafloor reflectivity *R*_sf_ = 0.13 is computed from the direct measurement of the rock properties of the IODP 504B borehole (Supplementary Fig. 1)^37^.

Guided by tracked reflector depths from the stacked seismic sections (Fig. 3), the corresponding events on the unstacked CMP gathers are traced automatically using the instantaneous phase from complex seismic trace analysis by Hilbert transforms^38^. The seismic attribute of instantaneous phase *α* highlights reflection continuity and has been successfully used for internal wave tracking on seismic images^39^. Here we first use the criterion of *cos*(*α*)=0.8 to contour the events and then pick their amplitudes on CMPs within the contour regions (Supplementary Fig. 3).

### MCMC method to search temperature contrast from reflectivity

The Monte Carlo Markov Chain (MCMC) algorithm is driven by estimates of the temperature and salinity contrasts (Δ*T*, Δ*S*) and their uncertainties inverted from seismic reflectivity^40^. During the MCMC process, the Markov chain length is set to *N* = 10,000; the first 500 iterations are ignored as the burn-in, during which the convergence should be quickly reached; and the rest of the chain is treated as an approximation of the true model space from which the estimates of the means, uncertainties, and marginal distributions are achieved. For the physical relation in the likelihood function, a simplified linearised approximation of the Zoeppritz equations^41^ is used because there are no shear waves in water:

2 $$R\left( \theta \right) = \frac{{\Delta \rho }}{{2\rho }} + \frac{1}{{2cos^2\theta }} \cdot \frac{{\Delta s}}{{2s}}$$

where $\Delta \rho = \rho _2 - \rho _1$, $\Delta s = s_2 - s_1$, $\rho = \left( {\rho _2 + \rho _1} \right)/2$, $s = \left( {s_2 + s_1} \right)/2$, $\theta = \left( {\theta _2 + \theta _1} \right)/2$, and $R\left( \theta \right)$ is the angle-dependent reflectivity. The subscripts 1 and 2 denote the layer above and below an interface. Instead of deriving contrasts of the P-wave velocity and in situ density of the water, we construct the relation from the *T-S* contrasts to the observed AVO responses using the equation of state of seawater^42^, i.e., $R\left( {\theta ,\Delta \rho ,\Delta s} \right) = R\left( {\theta ,\Delta T,\Delta S} \right)$, that directly provide the fundamental parameters of the seawater that control the acoustic speed *s* and density *ρ*, and therefore the reflection coefficient (Supplementary Fig. 4).

### Non-Simultaneous CTD Cast

One CTD cast labelled JC112–16 was deployed at the location of borehole ODP 504B (1°13.6′N, 83°43.9′W) by the RRS *James Cook* on 22 Dec 2014, i.e., 52 days ahead of the seismic acquisition. The principal water masses from shallow to deep: STUW, AAIW, and NPDW^43^ are identified. Physical parameters, such as acoustic speed, density ratio, and reflectivity, are calculated for matching the oceanographic parameters with the seismological parameters (Supplementary Fig. 2; some of them at 560 m depth are listed in Table 1). The density and acoustic speed at 560 m depth are used as the background values for calculating the reflectivity with Eq. (2). Potential temperature-salinity-density sections are presented from CTD casts along SAP_A, showing a lateral evenly layered temperature field and uniform water properties around 560 m (Supplementary Fig. 8).

### Near-Simultaneous Shipborne ADCP Data

In this study, the 75-kHz shipborne ADCP data recorded on the FS *Sonne*, which ran ~9 km (1 h) behind the seismic ship RRS *James Cook*, from 19:52 on 11 Feb 2015 to 11:55 on 15 Feb 2015, are used as near-concurrent data to extract the mean background current (Supplementary Fig. 5). The current profile has 80 depth bins with bin size of 10 m. The original ensemble interval is 60 s averaged from 12 pings. After the ship velocity correction using GPS data with the TRDI’s WINADCP software, the data are further smoothed using a sliding window of 30 ensembles to suppress the random noise. The data above ~650 m depth show a high coherence and are sufficient to derive the mean current around the target depth of 560 m. The average current profile from 0 m to 700 m depth is derived by averaging the whole dataset over the recording period (Fig. 1c). The mean values of (*u,v*) = (1.5 ± 0.2, 9.6 ± 1.2) cm/s in the depth range of 530–580 m is taken as the background current (Fig. 1a). And the mean vertical shear at this depth is (5.3±0.9)×10^-4^ s^-1^ (Table 1).

### Temperature contrast change rate

The time variation of $\Delta T$ within the seismic reflectors could be derived from the lateral temperature variation rate $\frac{\partial \Delta T }{\partial x}$and the lengthening rate $\frac{{dx}}{{dt}}$. Assuming the temperature contrast on the two-dimensional reflector is a function of *x*, *y* and *t*: $\Delta T = \Delta T(x,y,t)$, and $\Delta T$ does not change on the reflector (e.g., at the tip points), thus the material derivative of $\Delta T$ is zero:

3 $$\frac{{d\Delta T}}{{dt}} = 0 = \frac{{\partial \Delta T}}{{\partial t}} + u_R\frac{{\partial \Delta T}}{{\partial x}} + v_R\frac{{\partial \Delta T}}{{\partial y}}$$

where (*u*_*R*_,*v*_*R*_) is the horizontal expansion velocity of the reflector. A comparison of panels (b) and (c) in Fig. 4 suggests that $\frac{{\partial \Delta T}}{{\partial y}}$ is significantly smaller than $\frac{{\partial \Delta T}}{{\partial x}}$. The same figure also suggests that the seismic lines run close to the central part of the reflector and thus the expansion rate *v*_*R*_ in *y*-direction might be also smaller than *u*_*R*_ in *x*-direction. Therefore, the *y*-term in (3) can be reasonably neglected, and the formula (3) can be rewritten as:

4 $$\frac{{\partial \Delta T}}{{\partial t}} = - u_R\frac{{\partial \Delta T}}{{\partial x}}$$

where $u_R = \frac{{dx}}{{dt}}$ is the lengthening rate (expansion velocity in *x*-direction) of the reflector.

## Supplementary information

Supplementary Information

## Data Availability

Data are archived at the NERC’s British Oceanographic Data Centre and available on request from PI (R.W.H.). The final accepted version of this manuscript is available through Durham Research Online (dro.dur.ac.uk).

## Code availability

The MCMC algorithm used for temperature/salinity inversion from AVO data is available on request from Q.T.
