# Supplementary material for: Detecting changes at the leading edge of an interface between oceanic water layers
Source: Nat Commun. 2019 Oct 14;10:4674. doi: 10.1038/s41467-019-12621-8 (PMC6791866; doi:10.1038/s41467-019-12621-8)
Supplement: Supplementary file 1 — Supplementary Information [file 41467_2019_12621_MOESM1_ESM.pdf]

Supplementary materials for “Detecting changes at the leading edge of an interface between oceanic water layers”

Qunshu Tang*; Vincent C. H. Tong; Richard W. Hobbs; Miguel Morales Maqueda

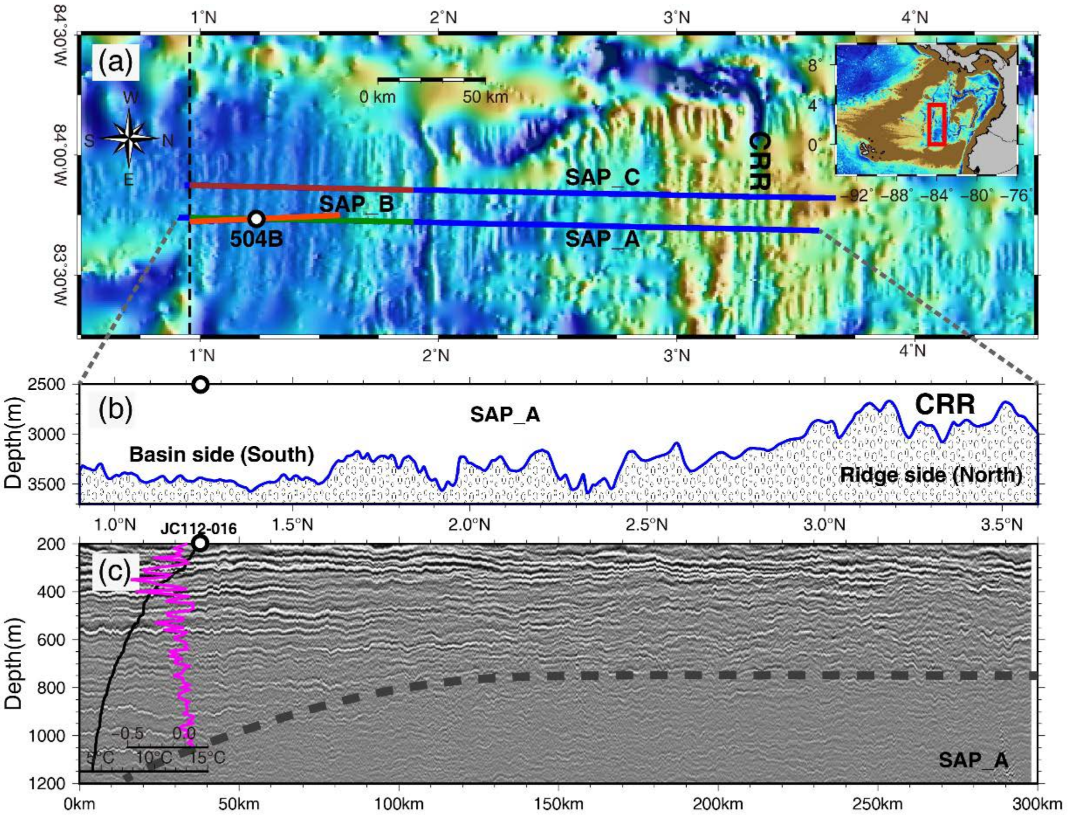

Supplementary Figure 1: Overview of the study region and the water column structure. (a) The location of seismic profiles SAP_A and SAP_C (blue lines). The sections of these profiles presented in this paper (green and brown respectively) and that of a third profile, SAP_B (orange) as shown in Figure 1. The black circle at the intersection of SAP_A and SAP_B is the site of ODP borehole 504B (Alt et al., 1996), which is also the location of CTD cast JC112-016 shown in Supplementary Figure 2. (b) The seafloor bathymetry along the section SAP_A. (c) Seismic image along SAP_A showing the water structure from the deeper basin to the shallower ridge (CRR: Costa Rica Ridge). The black and purple curves are the potential temperature and reflectivity ($\times 10^{-3}$) profiles computed from the CTD cast. Note distance along the seismic profiles is referenced to a common northing which is the dashed black line on (a).

The water at the basin (south) end of the profile is well stratified to a greater depth than at the ridge (north) end, underlain by the dashed line, where the water

layering is also more disturbed. The undulating reflections reveal how the stratification is differentially disturbed by the internal waves over the basin and over the ridge, indicating possible changes in the process of mixing along the profile. Excluding the effects of wind-driven mixing beneath the thermocline barrier in the eastern equatorial region (Sprintall & Cronin, 2001; Liu et al., 2016), a potential explanation for the change in reflectivity could be the upward emission and energy cascade of the internal tides generated over the shallower and rougher ridge topography, which is negatively correlated to the sub-thermocline stratification. The same phenomenon is also seen on SAP_C (not shown).

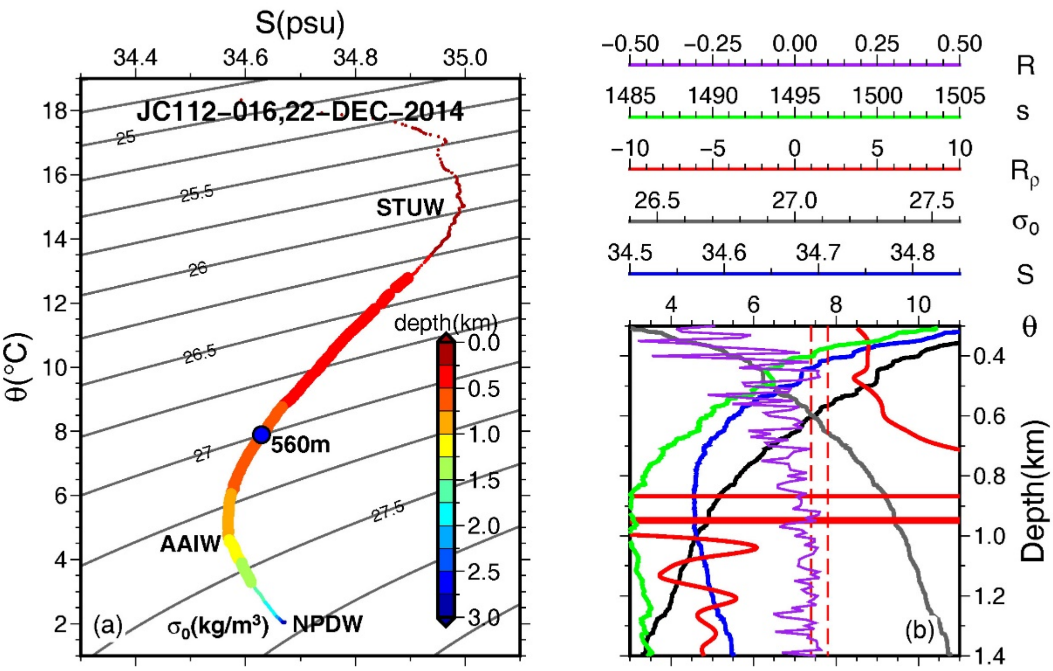

Supplementary Figure 2: The CTD cast (JC112-016) acquired on 22 Dec 2014 during cruise JC113 (~52 days prior to the seismic acquisition during cruise JC114) shows the general water properties at the intersection of SAP_A and SAP_B (Figure 1 and Supplementary Figure 1). (a) Potential temperature-salinity (T-S) diagram that is used to identify the water masses of the whole water column, with the colour scale denoting the water depth. The identified water masses are: STUW - Subtropical Underwater; AAIW - Antarctic Intermediate Water; and NPDW - North Pacific Deep Water. The thickened part shows the depth range between 300 to 1400 m, with the blue dot at the mean target depth of the reflector at ~560 m. Grey contours: potential density, σ_0, referenced to the surface. (b) Measured and estimated curves of potential temperature θ (°C, black), salinity S (psu, blue), potential density σ_0 referenced to 0 dbar (kg/m^3, grey), density ratio R_ρ (red), acoustic speed s (m/s, green), and reflectivity R (×10^{-3}, purple). Two red dashed lines mark the range of $1 < R_\rho < 2$, within which thermohaline staircases may exist.

Due to the time dependency of the finescale structure of the water, it is not possible to directly relate this CTD profile to the seismic images since they are not coincident in time. However, the hydrographic data do reveal the fundamental properties and stratification of the water column in the Panama Basin. For example, the *T-S* diagram shows the principal water masses from shallow to deep: STUW, AAIW, and NPDW (Fiedler & Talley, 2006); and the $R_\rho \approx 5$ at 560 m depth favours a double-diffusive stratification (Radko et al., 2014a). Reflectivity R is calculated form the hydrographic parameters (IOC et al., 2010).

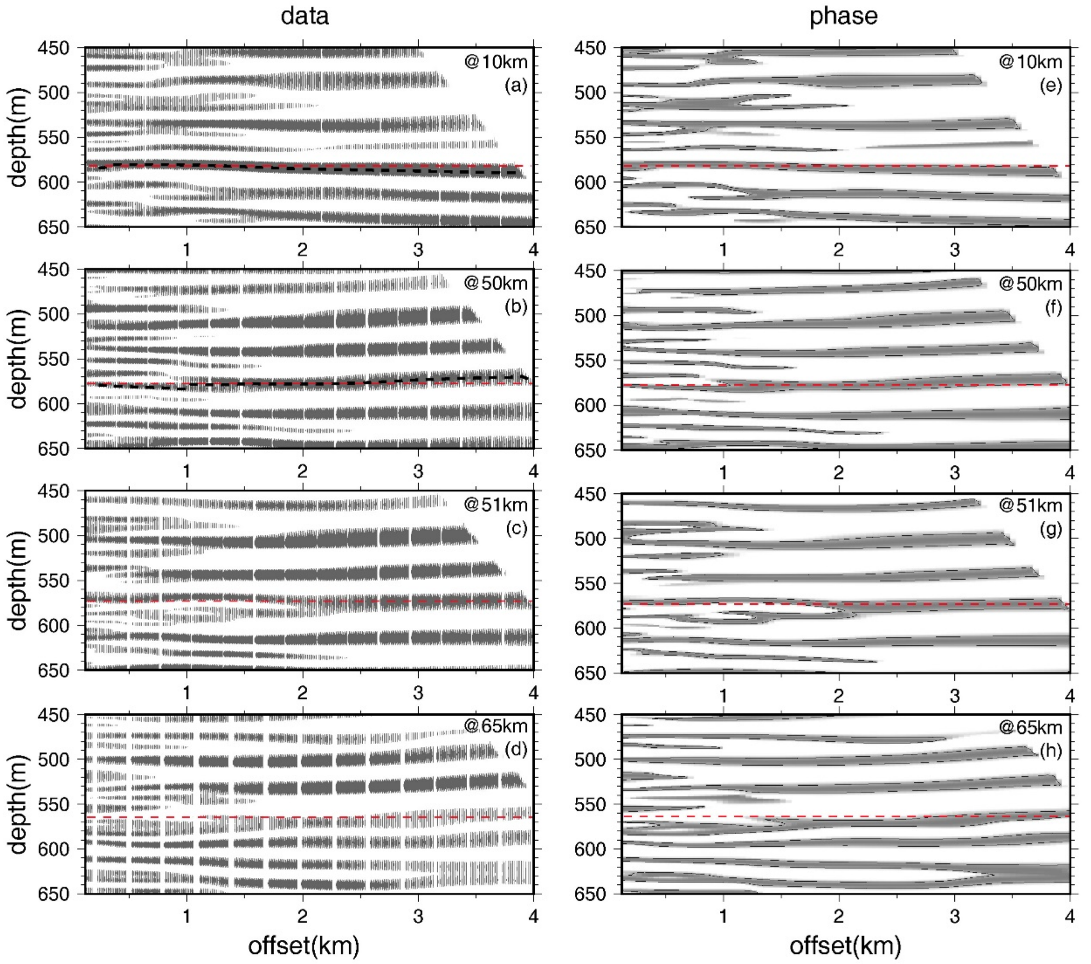

Supplementary Figure 3: Event tracking and AVO amplitude picking on the raw unstacked seismic data of common mid-point (CMP) super-gathers (Sheriff & Geldart, 1995; Paramo & Holbrook, 2005). The tracked events using the value 0.8 of the cosine of instantaneous phases (right column, black contours) of the corresponding data (left column) around the depths of the target reflections (red dashed lines). The AVO responses are then picked from the tracked events (black dashed lines). Examples from four representative locations of (a) 10 km, (b) 50 km, (c) 51 km, and (d) 65 km along SAP_B are presented. (a, e) The region with high reflectivity and coherent phase. (b, f) The end with weak reflectivity and still trackable phase. (c, g) The weak reflectivity but untrackable, bifurcated phase. (d, h) Neither the seismic

events nor the phase are identifiable far from the end of the target reflection.

AVO attributes are widely used to characterise physical properties across reflective boundaries in the solid Earth (Sheriff & Geldart, 1995). To date, the work by Paramo & Holbrook (2005) is the only study extending the AVO technique to quantify the temperature contrasts on seismic oceanography data. Here we improve this technique using an inversion procedure and apply it to a time-lapse seismic reflection to see the spatial-temporal temperature variations across a water sheet. Three steps are necessary to extract the AVO response (Methods): (1) true amplitude processing, (2) amplitude calibration, and (3) automatic amplitude picking. Four examples of event tracking are shown above. The contour of $cos(\alpha) = 0.8$ loses its lateral coherent at 51 km, where we define the tip of the reflector on raw unstacked CMP gathers. The trackable tips on CMP gathers are different than on the stacked seismic images (Figures 4 and 5) because of the enhanced signal-to-noise ratio on the stacked image.

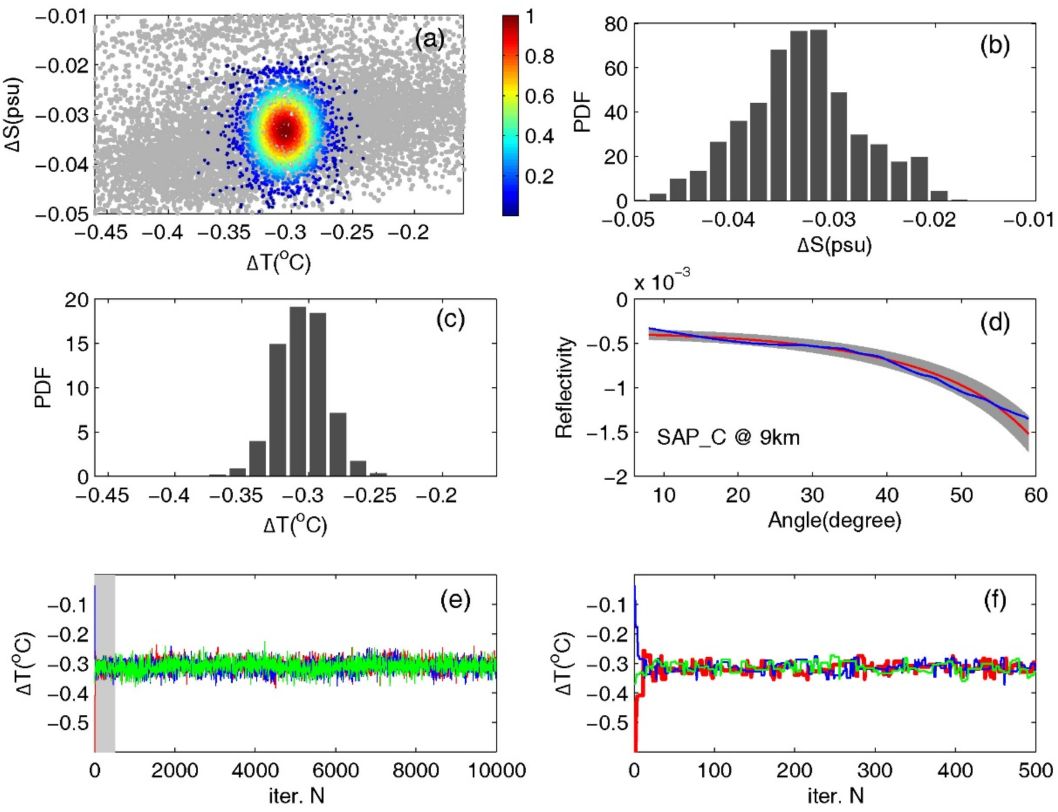

Supplementary Figure 4: An example at 9 km on SAP_C showing the derivation of the temperature contrast and its uncertainty using a Markov Chain Monte Carlo (MCMC) scheme based on Bayesian statistical inference (Tarantola, 2005). (a) Distributions of the rejected (grey dots) and accepted samples (coloured dots, normalised probability density function (PDF) of the bivariate Gaussian distribution fit). (b, c) Marginal distributions of salinity and temperature. (d) AVO responses of

the data (blue) and the prediction (red). The grey region is the 95% confidence interval of the fit. (e) Three runs of Markov chains with random initial starting models but showing the same convergence. The burn-in iterations (grey) are exaggerated in (f), showing the MCMC method has converged within 20 iterations. The contribution of salinity to the reflection coefficient is insignificant in the study region and hard to constrain, so we focus on the dominant temperature parameter as the primary cause of the observed reflectivity.

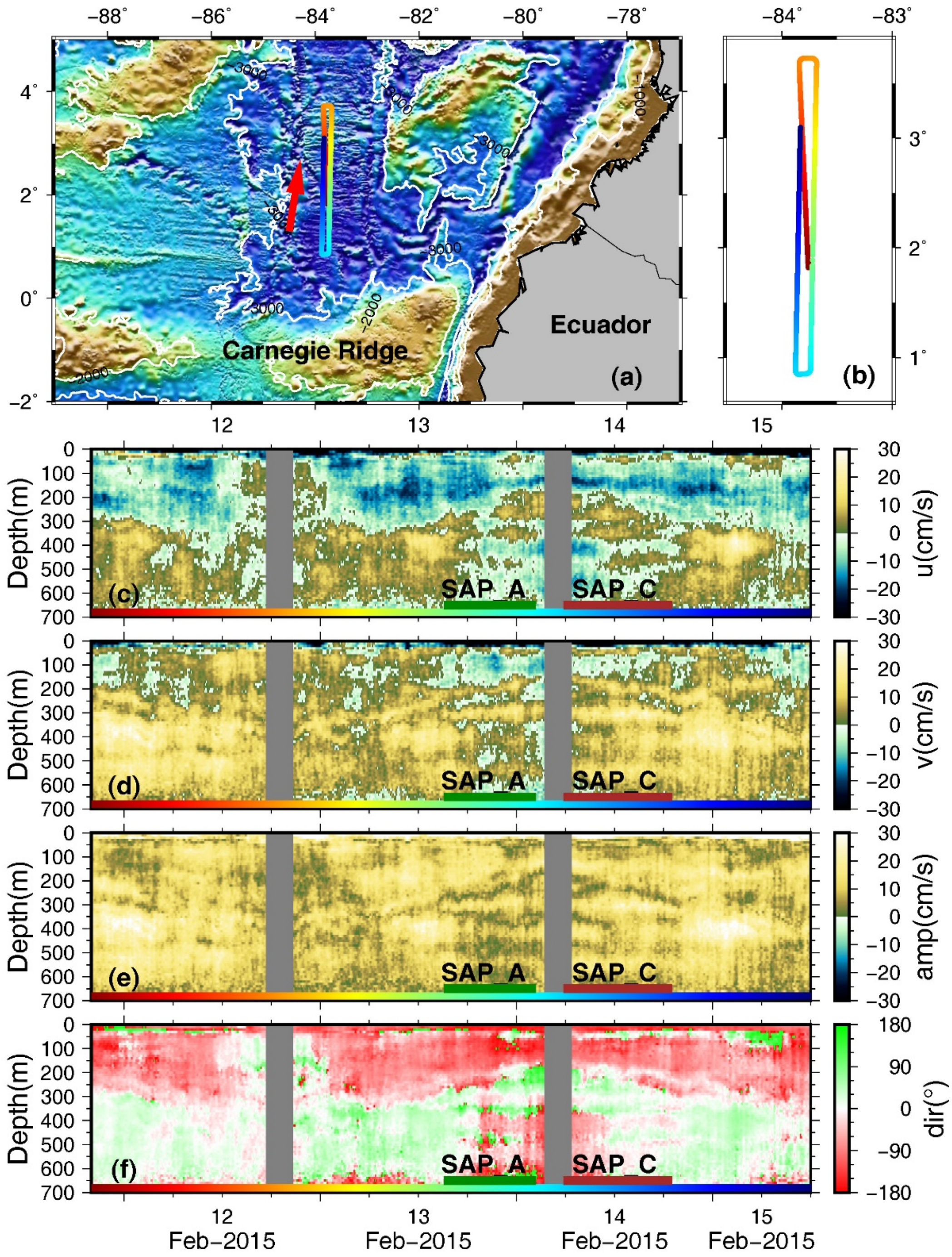

Supplementary Figure 5: Shipborne 75-kHz Acoustic Doppler Current Profilers (ADCP)

data recorded on FS *Sonne*, which sailed ~9 km behind (~1 hour delay) the RRS *James Cook* that was acquiring the seismic data. (a) The time of ADCP track, which is enlarged in (b), starts from dark red and ended at blue. Red arrow: mean flow direction at 560 m depth. (c, d) Eastward and northward current components u and v, respectively. The colour time scale used in (a) is shown along the bottom of these plots, as well as the acquisition periods of SAP_A (green) and SAP_C (brown). (e, f) The current strength and current direction with respect to north. The ADCP data at the turns are removed (shaded region).

To evaluate the development, e.g., lengthening, erosion, or strengthening, of the time-lapse reflections; the lateral advection caused by water current needs to be compensated for. Hence the mean current during the seismic acquisition was derived from shipborne ADCP data.

The average current profile shown in Figure 1c is calculated from these ADCP data. It can be seen that the large variations of current in both strength and direction are mainly above 350 m (thermocline). And below this depth (sub-thermocline), the velocity is more stable (< 12 cm/s) and the direction is uniform at around 10° from north. The eastward velocity component is relatively small and its contribution is estimated in Supplementary Figure 6. Tsuchiya & Talley (1998) suggest that the water at the target depth is sourced by the NNE-ward current bringing well-stratified water with layers and sheets into the central Panama basin water over the saddle area of the central Carnegie Ridge to the south.

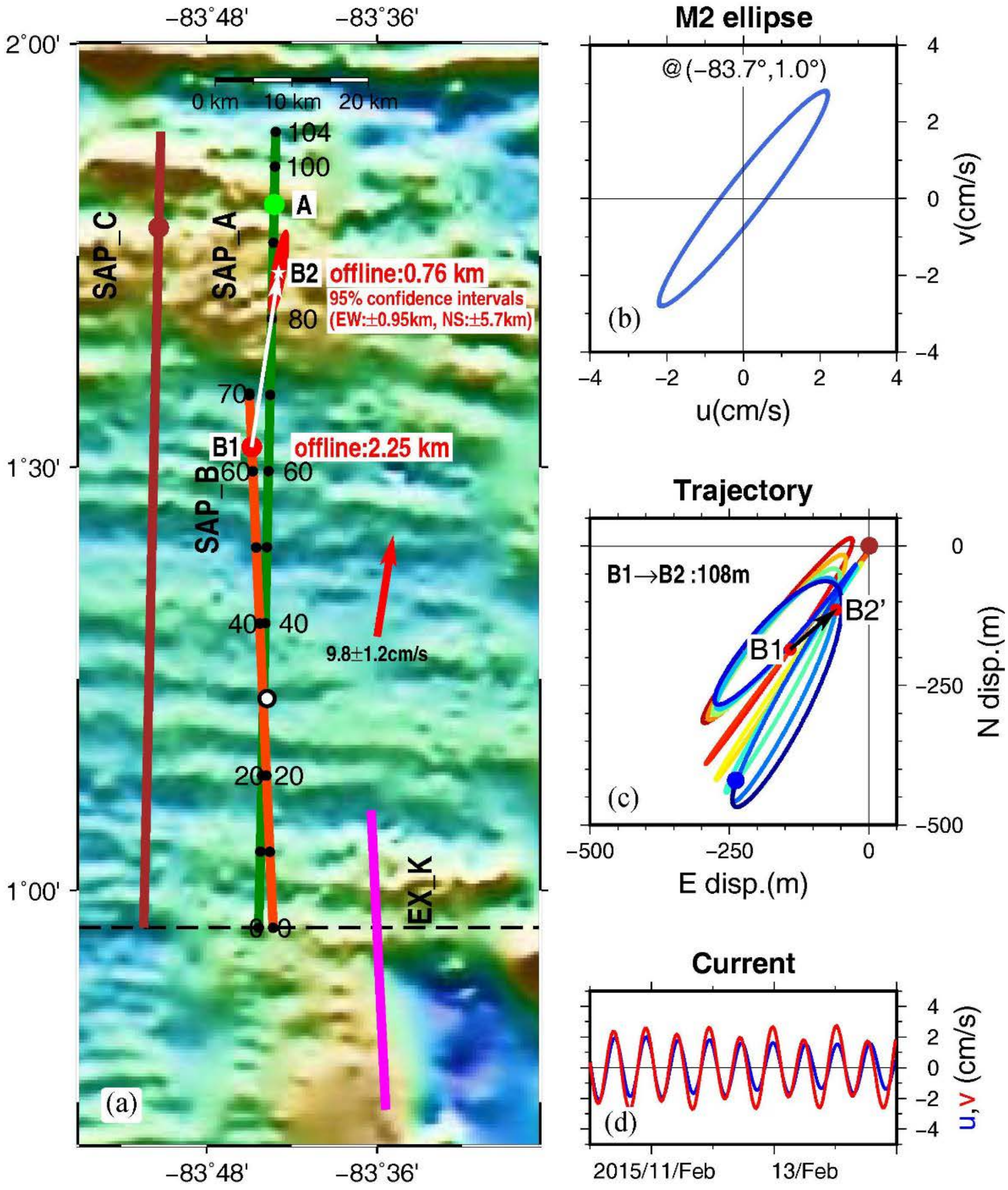

Supplementary Figure 6: (a) Measurement of the reflector's tip from line SAP_B to SAP_A: B1 (red dot) is the tip on SAP_B, B2 (white star) is the predicted parcel of B1 after 2.75 days when the tip A (green dot) on SAP_A was detected. The red oval region is the 95% confidence interval of B2 (EW: ±0.95 km, NS: ±5.7 km). The offline distance of B2 to line SAP_A is 0.76 km. Line EX_K (purple) is another line that clearly captured the same reflectors at depths of ~560 m and ~1000 m (not shown) on the same day before line SAP_B. (b) M2 tidal ellipse. (c) A parcel trajectory by tidal currents (d) of all available tidal constituents in the model TMD from 2015-02-10 00:00:00 (brown) to 2015-02-14 24:00:00 (blue). (d) East (u, blue) and north (v, red) components of the tidal current.

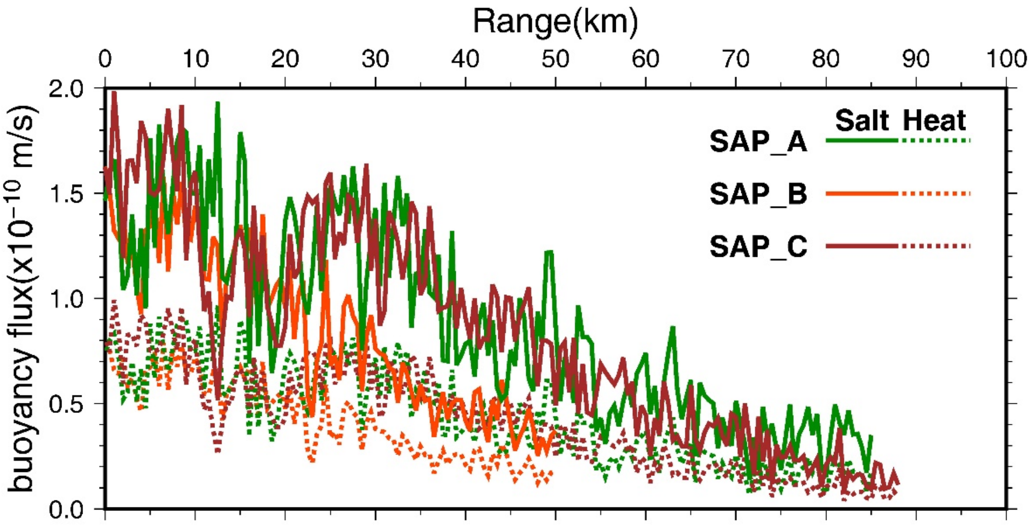

Supplementary Figure 7: Buoyancy fluxes for heat (a) and salt (b) across the interfaces on lines SAP_A (green), SAP_B (orange), and SAP_C (brown).

The empirical parametrisation scheme for a double-diffusive system of salt fingering based on the modified four-thirds flux-law is used to infer the heat and salt fluxes across the interface (Turner, 1965; Schmitt, 1979; Shen, 1993; Radko et al., 2014b). Since ΔT is much better constrained than ΔS in our results, the parametrisation is modified using only ΔT to compute the buoyancy fluxes:

$$\begin{cases} \alpha F_T = C(gk_T)^{\frac{1}{3}}\left(\frac{\alpha\Delta T}{R_\rho}\right)^{4/3} \\ \beta F_s = \alpha F_T/R_f \\ R_\rho = \frac{\alpha\Delta T}{\beta\Delta S} \\ R_f|_{R_\rho\simeq 5} = 0.5 \\ C = 0.05 + 0.3R_\rho^{-3} \end{cases} \tag{S1}$$

where the notations are: F_T, flux of temperature across an interface; F_s, flux of salt; R_f, buoyancy flux ratio of heat αF_T to salt βF_s; R_ρ, density ratio; α and β, heat expansion and salt contraction coefficients; C, flux-law coefficient; g, acceleration due to gravity; and k_T, effective thermal diffusivity.

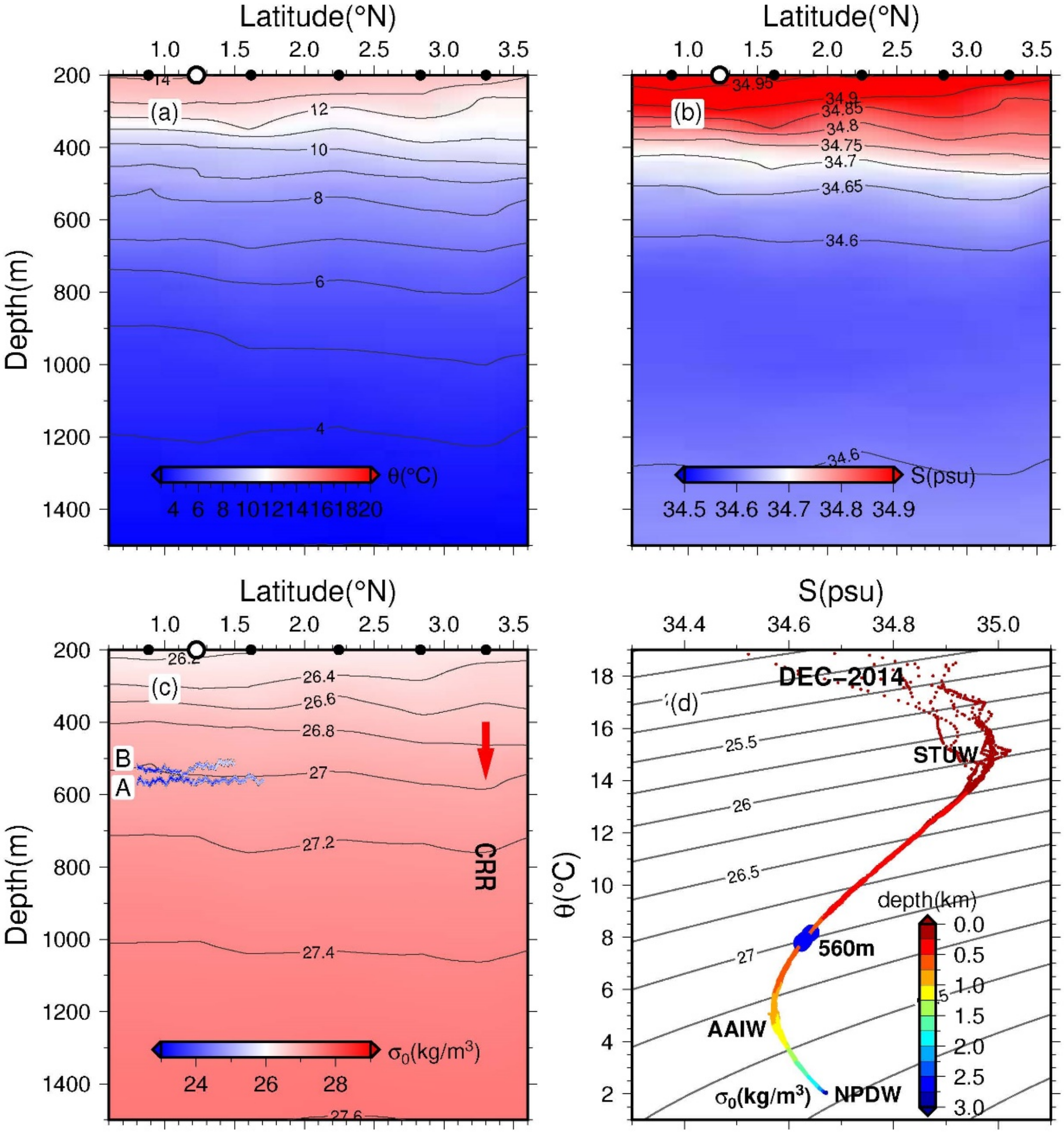

Supplementary Figure 8: Temperature (a), salinity (b), and potential density (c) sections from the CTD casts acquired in Dec-2014 along seismic line SAP_A. The tracked reflection B (on SAP_B, with upward shift of 50 m) and reflection A (on SAP_A) are superimposed on (c). CRR (Costa-Rica Ridge). (d) Water properties of all CTD casts. See annotations for Supplementary Figure 2.

Supplementary References:

Alt, J. C., Kinoshita, H., Stokking, L. B., & Michael, P. J. (1996), *Proc. ODP, Sci. Results, 148*, College Station, TX (Ocean Drilling Program), doi:10.2973/odp.proc.sr.148.1996.

Fiedler, P. C., & Talley, L. D. (2006), Hydrography of the eastern tropical Pacific: A review, *Progress in Oceanography*, *69*(2-4), 143-180, doi:10.1016/j.pocean.2006.03.008.

IOC, SCOR, & IAPSO (2010), *The International Thermodynamic Equation of Seawater – 2010: Calculation and use of thermodynamic properties*, 196 pp., Intergovernmental Oceanographic Commission, Manuals and Guides No. 56, UNESCO.

Liu, C. Y., Kohl, A., Liu, Z. Y., Wang, F., & Stammer, D. (2016), Deep-reaching thermocline mixing in the equatorial pacific cold tongue, *Nature Communications*, *7*, 11576, doi:10.1038/ncomms11576.

Paramo, P., & Holbrook, W. S. (2005), Temperature contrasts in the water column inferred from amplitude-versus-offset analysis of acoustic reflections, *Geophysical Research Letters*, *32*, L24611, doi:10.1029/2005gl024533.

Radko, T., Bulters, A., Flanagan, J. D., & Campin, J. M. (2014a), Double-diffusive recipes. Part I: Large-scale dynamics of thermohaline staircases, *Journal of Physical Oceanography*, *44*(5), 1269-1284, doi:10.1175/JPO-D-13-0155.1.

Radko, T., Flanagan, J. D., Stellmach, S., & Timmermans, M. L. (2014b), Double-diffusive recipes. Part II: Layer-merging events, *Journal of Physical Oceanography*, *44*(5), 1285-1305.

Schmitt, R. W. (1979), Flux Measurements on Salt Fingers at an Interface, *Journal of Marine Research*, *37*(3), 419-436.

Shen, C. Y. (1993), Heat-Salt Finger Fluxes across a Density Interface, *Physics of Fluids a-Fluid Dynamics*, *5*(11), 2633-2643, doi:Doi 10.1063/1.858727.

Sheriff, R. E., & Geldart, L. P. (1995), *Exploration Seismology*, 2nd ed., 592 pp., Cambridge Univ. Press, Cambridge, U. K., ISBN:0521462827.

Sprintall, J., & Cronin, M. F. (2001), Upper ocean mixing processes, *Encyclopedia of Ocean Sciences, Academic Press*, *6*, 3120-3128.

Tarantola, A. (2005), *Inverse problem theory and methods for model parameter estimation*, 342 pp., Soc. for Ind. and Appl. Math., Philadelphia, Penn.

Tsuchiya, M., & Talley, L. D. (1998), A Pacific hydrographic section at 88 degrees W: Water-property distribution, *Journal of Geophysical Research-Oceans*, *103*(C6), 12,899-812,918.

Turner, J. S. (1965), The coupled turbulent transports of salt and and heat across a sharp density interface, *International Journal of Heat and Mass Transfer*, *8*(5), 759-767, doi:10.1016/0017-9310(65)90022-0.
